# The Life Course Origins of the Race Disparity in Cognitive Functioning in Brazil

**DOI:** 10.1093/geroni/igab046.2275

**Published:** 2021-12-17

**Authors:** Mateo Farina

**Affiliations:** University of Southern California, Los Angeles, California, United States

## Abstract

Background: Cognitive health is a major concern for understanding population health in Brazil. Race inequalities have been found for several health outcomes but less is known about older adult cognitive health. Health inequalities have been tied to several life course factors, but less is known about how the racial stratification in Brazil may contribute to race disparities in cognitive health. Method: Data come from the Brazilian Longitudinal Study of Aging. We used nested regression models to examine the life course origins of the race differences in cognitive functioning. Results: Whites had better cognitive functioning than non-Whites. Education reduced these differences by about half. Health behaviors and cardiometabolic conditions had little to no impact. Discussion: Race differences in cognitive functioning in Brazil are in large part attributable to educational opportunities. These finding point to the importance of cognitive development in childhood to understand racial disparities in later life cognitive health.

